# Enhanced potent immunosuppression of intracellular adipose tissue-derived stem cell extract by priming with three-dimensional spheroid formation

**DOI:** 10.1038/s41598-024-59910-x

**Published:** 2024-04-20

**Authors:** Witchayapon Kamprom, Rattanawan Tangporncharoen, Nuttapoom Vongthaiwan, Patcharapa Tragoonlugkana, Jitrada Phetfong, Chatchai Pruksapong, Aungkura Supokawej

**Affiliations:** 1 Department of Clinical Microbiology and Applied Technology, Faculty of Medical Technology, Mahidol University, Nakhon Pathom, Thailand; 2 Department of Clinical Microscopy, Faculty of Medical Technology, Mahidol University, 999 Phutthamonthon Sai 4, Salaya, Phutthamonthon, Nakhon Pathom, 73170 Thailand; 3 Department of Surgery, Phramongkutklao Hospital and College of Medicine, Bangkok, Thailand

**Keywords:** Adipose tissue-derived stem cell, Spheroid, Intracellular extract, Immunomodulation, Cell-free product, Cell biology, Immunology, Stem cells

## Abstract

Immunomodulatory properties of mesenchymal stem cells are widely studied, supporting the use of MSCs as cell-based therapy in immunological diseases. This study aims to generate cell-free MSC extract and improves their immunomodulatory potential. Intracellular extracts were prepared from adipose-derived stem cells (ADSC) spheroid via a freeze-thawing method. The immunomodulatory capacities of ADSC spheroid extracts were investigated in vitro, including lymphocyte proliferation, T regulatory cell expansion, and macrophage assays. A comparative study was conducted with ADSC monolayer extract. The key immunomodulatory mediators presented in ADSC extract were identified. The results revealed that ADSC spheroid extract could suppress lymphocyte activation while enhancing T regulatory cell expansion. Immunomodulatory molecules such as *COX-2*, *TSG-6*, and *TGF-β1* were upregulated in ADSC priming via spheroid culture. Selective inhibition of COX-2 abrogates the effect of ADSC extract on inducing T regulatory cell expansion. Thus, ADSC spheroid extract gains high efficacy in regulating the immune responses which are associated in part by COX-2 generation. Furthermore, ADSC spheroid extract possessed a potent anti-inflammation by manipulation of TNF-α production from LPS-activated macrophage. Our current study has highlighted the opportunity of using cell-free extracts from adipose tissue-derived mesenchymal stem cells spheroid as novel immunomodulators for the treatment of immunological-associated diseases.

## Introduction

Mesenchymal stem cells (MSCs) play a key role in immunomodulation, which impacts both innate and adaptive immune responses. Together with hypoimmunogenic capacity, MSCs have been regarded as therapeutic agents in the treatment of immune-mediated diseases and tissue repair. There are increasing reports of clinical uses of MSCs in patients with inflammatory disease, autoimmune disease, and graft-versus-host disease^1^. The therapeutic effects of MSCs are at least partially mediated through the paracrine mechanism. It is well-documented that MSCs secrete several bioactive molecules involved in promoting angiogenesis, anti-apoptosis, anti-fibrosis, and immunomodulation^2,3^. MSCs conditioned medium and microvesicles are known to mediate immunosuppressive functions and induce anti-inflammation^4,5^. Hence, biological factors produced by MSCs raise the opportunity of using cell-free MSCs for therapeutic uses. Among stem cell-free derivatives, the cellular extract is easier to prepare and contains abundant intracellular proteomes that play roles in anti-aging, angiogenesis, wound healing, and regulating cellular activities^6–9^. However, there is a lack of study on the role of MSC cellular extract in controlling immune cell functions. Interestingly, a previous study demonstrated that apoptotic MSCs exhibited immunosuppressive ability and were able to alleviate graft-versus-host disease (GvHD)^10^. Thus, we hypothesized that the extraction of intracellular components from MSCs may be a valuable source for the generation of immunomodulatory molecules.

The immunosuppressive abilities of MSCs are plasticity which exhibits both stimulation and suppression of the immune response. The beneficial effects of naïve MSCs in pre-clinical and clinical studies have been reported the controversial results, therefore limiting their therapeutic efficiency^11,12^. Priming naïve MSC with either inflammatory cytokines such as IFN-gamma and TNF-alpha or culturing under hypoxia, could improve its immunomodulation capacities. A three-dimensional (3D) culture system is a simple self-activation approach. MSCs culturing as spheroid enhanced cell survival, cytokine production, and differentiation potential^13,14^. Moreover, MSC spheroids provide a homogenous cell population and promote the secretion of several immunomodulatory factors^15^, but the roles of intracellular MSC spheroid extract have not been clarified. We anticipate that culturing MSCs in a 3D spheroid system potentially modulates MSC phenotype and leads to alteration of immunoregulatory effects.

Based on the abovementioned, this study aims to enhance the immunomodulatory effects of intracellular MSC extracts. To achieve the goal, adipose tissue-derived stem cells (ADSCs) were cultured as spheroids using low-binding plate techniques. The intracellular contents of ADSC spheroids and ADSC monolayers were extracted through a freeze-thawing method. The immunomodulatory effects of ADSC extracts were investigated, including lymphocyte proliferation, T regulatory cell population, and anti-inflammation. Alteration in gene expression profile in ADSC spheroid was examined in comparison to conventional ADSC monolayer. Furthermore, the candidate immunomodulatory molecules were identified. The knowledge gained from this study would address the limitation of MSC monolayer and clarify an unclear immunomodulatory mechanism of MSCs. Moreover, the successful generation of MSC spheroid extracts with high immunomodulation efficiency will lead to the development of novel biological products for the treatment of immunological-associated diseases. Based on technology and innovation nowadays, there is the possibility to generate multiple MSC spheroids and produce large amounts of off-the-shelf MSC products for clinical uses in the future.

## Methods

### Adipose tissue-derived stem cell culture

Mesenchymal stem cells were isolated from adipose tissue. The procedure was approved by the ethical committee for human research of the Mahidol University Central Institutional Review Board in accordance with the Declaration of Helsinki, The Belmont Report, CIOMS Guidelines, and the International Conference on Harmonization in Good Clinical Practice (MU-CIRB 2021/074.2603). Briefly, lipoaspiration was taken from healthy donors who already signed the informed consent. Adipose tissues were digested with 0.025% type I collagenase (Worthington Biochemical Corporation, USA) at 37 °C for an hour with shaking. The cells were cultured in complete medium containing Dulbecco’s Modified Eagle Medium low glucose (DMEM-LG, Gibco, USA), 10% (v/v), fetal bovine serum (Sigma-Aldrich, USA), 1% penicillin–streptomycin (Gibco, USA), and 1% GlutaMAX (Gibco, USA) at 37 °C in humidified atmosphere containing 5% CO_2_. After 48-h incubation, the non-adherent cells were removed. The adherent cells were further cultured until the cell reached 80% confluence. Adipose tissue-derived cells were assessed for mesenchymal stem cell characteristics. Adipose tissue-derived stem cells (ADSCs) at passages 3–6 were used in spheroid formation.

### Adipose tissue-derived stem cell characterization

To determine MSC immunophenotype, the cells were stained with anti-CD73, anti-CD105, anti-CD90, anti-CD45, and anti-CD34 (List of antibodies shown in supplementary data, Table S1). The expression of those markers was analyzed by FACS Canto II and FACSDiva software (BD bioscience, USA).

The multilineage differentiation of ADSCs was investigated by culturing the cells under a specific induction medium. For osteogenic differentiation, ADSCs were cultured in osteogenic induction medium containing complete DMEM-LG medium, 10 mM β-glycerophosphate, 0.1 μM dexamethasone, and 50 μg/ml ascorbic acid for 21 days. Accumulations of calcium deposition in osteoblastic cells were determined by Alizarin Red S (Sigma-Aldrich, USA) staining. To induce adipocyte differentiation, the cells were maintained in an adipogenic induction medium for 14 days. Fat droplet containing adipocyte was determined by Oil Red O (Sigma-Aldrich, USA) staining.

### Generation of three-dimensional spheroids

To form spheroids, passages 3–6 of ADSCs were harvested and plated in a low binding 96-well plate with round bottom (Corning, USA) at a cell density of 2500 cells/cm^2^ in a small amount of complete DMEM medium^16^. Briefly, 25,000 ADSCs were resuspended in 50 μl of complete DMEM medium and seeded in each well of a low binding plate to form 25 k spheroid. For the preparation of ADSC spheroid extract, at least 96 spheroids were required. The cells were cultured at 37 °C in a humidified atmosphere containing 5% CO_2_ for 6 days without medium change. Change in cell morphology was observed under an inverted microscope (Olympus, Japan) throughout the culture period. The diameter of day 6 spheroids was analyzed using ImageJ software (NIH). On day 6 of cultivation, all spheroids were collected with the culture medium and transferred to a 15-ml centrifuge tube. The spheroids were allowed to set at the bottom of a tube for 5 min and then the supernatant was aspirated. To eliminate the residual fetal bovine serum, the spheroids were washed with PBS using a similar procedure as the culture medium removal. Then, the spheroids were dissociated by trypsin treatment for extraction of intracellular components and for flow cytometry analysis. To dissociate the spheroids, 0.25% Trypsin/EDTA (Gibco, USA) was added at a volume of 1 ml per 30 spheroids without mixing^17^. The suspension was incubated at 37 °C for 5 min and then mixed up and down until no cell clumping was observed followed by adding complete DMEM medium to inactivate trypsin activity. After cell dissociation, a trypan blue exclusion assay was performed to determine whether the cell remains viable. The cell pellet was resuspended in an appropriate volume of complete DMEM medium and count for viable cells by mixing with 0.4% trypan blue solution at 1:1 ratio and counting with a hemocytometer. In addition, apoptotic cells throughout the spheroid were investigated using FITC Annexin V apoptosis detection kit (BD Biosciences, USA).

### Preparation of ADSC spheroid extracts

The intracellular contents of ADSC spheroids were extracted using a simple freezing–thawing approach^18–21^. ADSC spheroids were harvested on day 6 of incubation. Herein, the size of 25 k spheroid used for cell extract preparation was in range of 0.547 ± 0.006 to 0.774 ± 0.097 mm to avoid cell dead in the central region. The compact ADSC spheroids were dissociated into single cells to facilitate complete cell disruption as mentioned above. After cell counting, a spheroid obtained viable single cells at approximately 16,218 ± 1,942 cells/spheroid (n = 9). The cells were resuspended in 0.9% normal saline (NSS) at a density of 10 million viable cells/ml. The cell suspension was lysed by rapidly frozen at − 80 °C overnight and thawed at 37 °C for 10 min. The freeze-thawing was repeated for three cycles. The crude debris was discarded by centrifugation at 14,000 g, 4 °C for 15 min. Finally, the supernatant was collected, and stored at − 80 °C until use (no longer than 1 month). The total protein concentration was measured using BCA protein assay (Abcam, UK). Additionally, the presence of cell debris and nuclear fragments in the cellular extracts was investigated by propidium iodide (BD Biosciences, USA) staining followed by flow cytometry analysis to ensure complete cell lysis (Supplementary data, Fig. S1).

### Quantitative real-time PCR

Total RNA was extracted using Trizol (Invitrogen Corporation, USA). RNA was converted to cDNA using iScript™ Reverse Transcription Supermix (Bio-Rad, USA). For quantitative real-time PCR, the forward and reverse primers (10 μM each) of interested genes (List of oligoes primers shown in supplementary data, Table S2) were mixed with nuclease-free water, KAPA SYBR Fast qPCR master mix (KAPA biosystem, USA), and cDNA. The qPCR was performed by CFX96™ Real-Time PCR detection system (Bio-Rad, USA). The difference in mRNA expression levels was assessed by normalization of the target gene with an internal reference gene, GAPDH, and calculated as a comparative C_T_ method (2^−∆C^_T_).

### Effect of ADSC spheroid extracts on suppression of lymphocyte proliferation

Peripheral blood was taken from healthy donors who already signed the informed consent. The procedure was approved by the ethical committee for human research of the Mahidol University Central Institutional Review Board (MU-CIRB 2021/074.2603). Peripheral blood mononuclear cells (PBMCs) were separated via ficoll-hypaque centrifugation. To investigate the effect of ADSC spheroid extracts and ADSC monolayer extract on suppression of lymphocyte proliferation, PBMCs were stimulated with 1% phytohemagglutinin (PHA) (Gibco, USA), a T cell mitogen and were cultured in the presence or absence of ADSC spheroid extract at final concentration of 0, 25, 50, 100, and 200 µg/ml. The unstimulated PBMCs were used as a negative control. After 3 days of incubation, the changes in the number of living lymphocytes were monitored using cell counting kit 8 (Sigma-Aldrich, USA). Optical density (O.D.) was read at 450 nm. The data was presented as optical density and the stimulation index by calculation (O.D. values of test/O.D. values of unstimulated control).

### T regulatory cell analysis

To investigate the effect of ADSC spheroid extracts and ADSC monolayer extract on induction of T regulatory cell subpopulation, 1 × 10^6^ PBMCs were stimulated with 1% PHA and exposed to either 100 µg/ml ADSC spheroid extract or 100 µg/ml ADSC monolayer extract. The PHA-stimulated PBMC alone was used as a control. After 72 h of incubation, the cells were harvested and stained with a Treg detection kit (Miltenyi Biotec, Germany) (List of antibodies shown in Supplementary data, Table S1). The T regulatory cell subpopulation was assessed by flow cytometry (BD bioscience, USA) based on the CD3^+^CD4^+^CD25^+^FoxP3^+^ cell population.

### COX-2 inhibitory effects

For studying the selective COX-2 inhibitory effects, 10 μM celecoxib (Sigma-Aldrich, USA) was added in culture condition together with 100 μg/ml ADSC spheroid extracts or ADSC monolayer extract as mentioned above. The PBMCs cultured without selective COX-2 inhibitor treatment were used as the untreated control. The effects of COX-2 inhibition on the proliferation capacity of PHA-stimulated PBMCs were evaluated by cell counting kit (CCK8) assay. Meanwhile, the inhibitor effects on the induction of T regulatory cells by ADSC spheroid extract were investigated via flow cytometry analysis.

### Western blotting analysis

An equal amount of ADSC spheroid extract and ADSC monolayer extract (15 μg) were loaded in each lane of a 12.5% SDS-PAGE and subjected to electrophoresis. The separated proteins were transferred to a polyvinylidene difluoride (PVDF) membrane (Merck Millipore) using the Mini-Protean© system (Bio-Rad Laboratories, Germany). To block the non-specific binding site, the membrane was incubated in 5% non-fat milk in TBST for an hour followed by incubation with anti-COX-2 (#12282, Cell signaling technology, USA) and anti β-actin (MAB1501, Merck, Germany) at 4 °C for overnight. After incubation, the membrane was washed three times with TBST and incubated with secondary antibody conjugated HRP for 1 h at room temperature. For western blotting detection, the blot was incubated with an ECL reagent (GE, UK) and exposed using the ChemiDoc™ MP imaging system (Bio-Rad Laboratories, USA) to detect the signal. The intensity of bands was evaluated by Image Lab software (Bio-Rad Laboratories, USA) and presented as relative intensity to β-actin.

### ELISA assay

The presence of TSG-6 and TNF-α was measured by ELISA assay (ELK biotechnology, USA). ADSC spheroid extract, ADSC monolayer extract, and supernatant of THP-1 were collected and kept at − 20 °C until use. ELISA was performed according to the manufacturer’s instructions. The samples (100 μl each) were incubated with a pre-coated plate for 80 min at 37 °C followed by biotinylated antibody, streptavidin-HRP, and TMB substrate, respectively. The absorbance was measured at 450 nm with correction. The concentration of target proteins was calculated according to the standard curve.

### In vitro modulation of macrophage

To investigate the effects of ADSC spheroid extracts on the modulation of macrophage, THP-1 (ATCC, USA), a monocyte cell line was activated with 5 nM PMA (Peprotech Asia, Israel) to prepare cells toward the M0 stage. After activation, M0 macrophages were cultured in the presence of 100 ng/ml LPS (Sigma-Aldrich, USA) for 90 min. to induce cell differentiation followed by incubation with either ADSC spheroid extract or ADSC monolayer extract for a further 24 h. M0 macrophage cultured in complete RPMI (Gibco, USA) alone served as control. The macrophage phenotypes were characterized. The expression of M1-associated genes (*TNF-α, IL-1β,* and *CCR7*) and M2-associated genes (*CD206*, *TGM*, and *DC-SIGN*) were investigated by quantitative real-time PCR. Besides, secretion levels of TNF-α by macrophage were investigated by ELISA assay (ELK biotechnology, USA).

### Statistical analysis

Data were presented as the mean ± standard error of the mean (SEM) of at least 3 individual experiments. To assess the significance of differences between observed data, the data were analyzed by Mann–Whitney U test, Pair *t-*test, and one-way ANOVA (GraphPad Prism software, USA). The *P*-value less than 0.05 was considered to be statistically significant.

### Ethics approval and consent to participate

Human adipose tissue-derived stem cells were isolated from adipose tissues. The lipoaspiration was taken from healthy donors who already signed the informed consent. Human mononuclear cells were isolated from peripheral blood of healthy donors. Written informed consent was obtained from all donors prior to collection. All procedures were approved by the ethical committee for human research of the Mahidol University Central Institutional Review Board in accordance with the Declaration of Helsinki, The Belmont Report, CIOMS Guidelines, and the International Conference on Harmonization in Good Clinical Practice. Project title: A pilot study of biologics derived from mesenchymal stem cells in bone regeneration and anti-inflammation. Approved number: MU-CIRB 2021/074.2603. Approved date: March 26th, 2021.

## Results

### Generation and characterization of adipose tissue-derived stem cell spheroids

**Figure 1:**
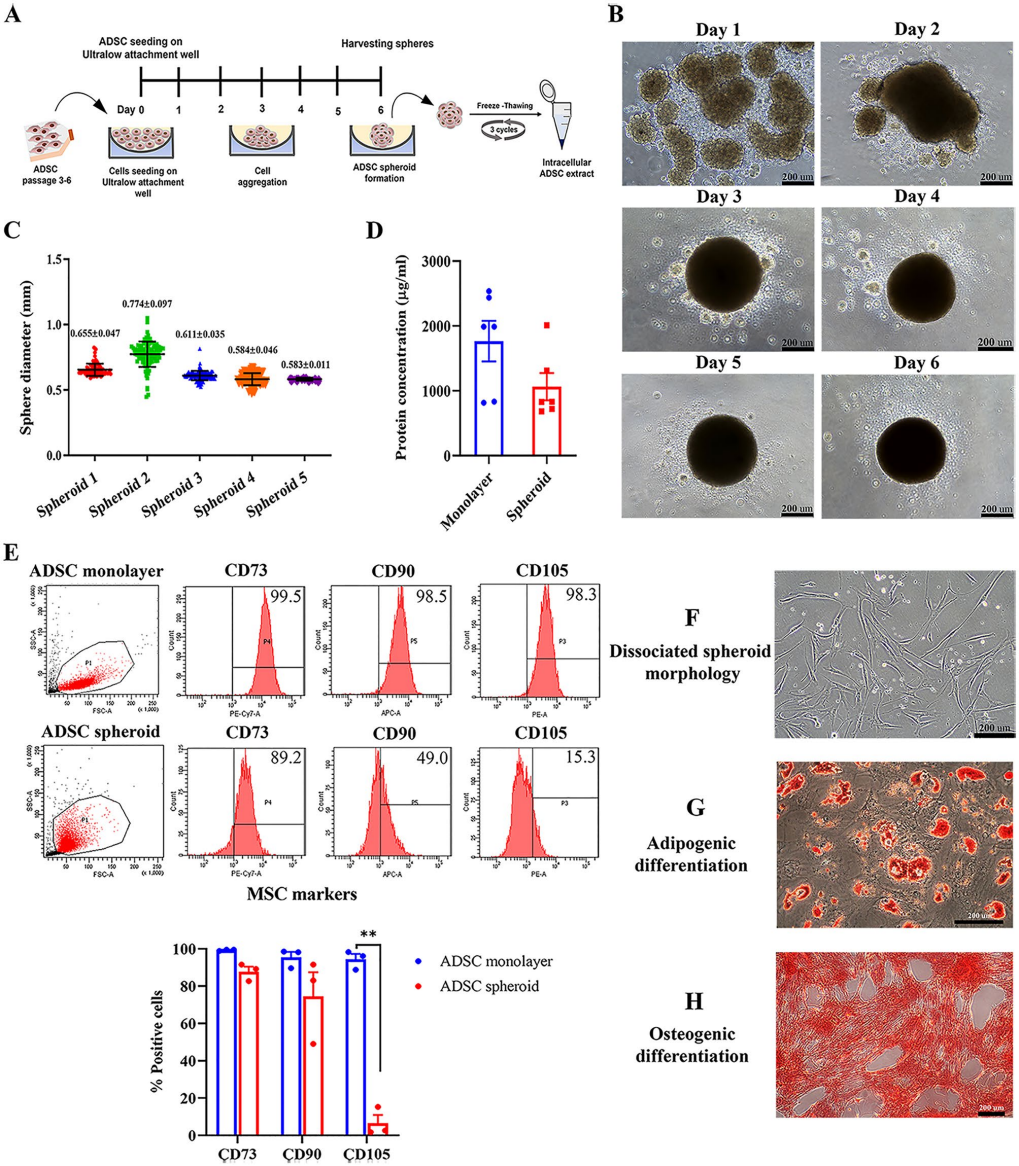
Spheroid formation and characterization. (**A**) Schematic illustration of spheroid formation in a low-binding plate and the extraction of intracellular ADSC spheroid contents. (**B**) The morphological changes of ADSCs which were seeded in Ultralow attachment wells for 6 days. (**C**) Analysis of spheroid size distribution by NIH Image J software from 5 ADSC donors and presented as mean ± SEM (*n* = 96 for each ADSC donor). (**D**) Protein measurement of intracellular extract derived from ADSC spheroids and ADSC monolayer. Data is presented as mean ± SEM (*n* = 6). (**E**) Immunophenotype of ADSC spheroids for CD90, CD73, CD105, CD34, and CD45 compared to ADSC monolayer. The scatter plot represents one out of three ADSC cases. The percentage of CD73, CD90, and CD105 positive cells obtained from ADSC spheroids and ADSC monolayer were compared using Pair *t*-test (n = 3). *P < 0.05; **P < 0.01 (**F**) Morphology of dissociated ADSC spheroid after replating in a polystyrene culture plate. (**G**, **H**) Adipogenic and osteogenic differentiation capacities of dissociated ADSC spheroids after cultured in adipogenic- and osteogenic-induction medium. The scale bar is 200 μm.

All the ADSCs used for the spheroid formation met the minimal criteria for defining MSCs as proposed by the International Society for Cellular Therapy^22^ (Supplementary data, Table S3 and Fig. S2). Adipose tissue-derived mesenchymal stem cells (ADSCs) were cultured in the low-binding plate for 6 days to generate the spheroids. After spheroid harvesting, the intracellular components of ADSC spheroids were extracted by a freeze-thawing method (Fig. 1A). The cultured ADSCs at 25,000 cells/spheroid demonstrated that there was no attachment of ADSCs after cell seeding, therefore the cells were forced to interact with each other. On day 1, ADSCs began to form multiple small clumps followed by self-assembling into a single irregular-shaped aggregate. The spherical-shaped aggregate appeared around day 3 of culture (Fig. 1B). The spheroid progressively compacted and provided the smallest size on day 6. A spheroid diameter average was 0.645 ± 0.091 mm. (Fig. 1C). The spheroids were harvested and assessed for cell viability. There was no significant difference in the number of viable cells in the ADSC spheroid compared with the ADSC monolayer (Supplementary data, Fig. S3A–C). This agrees with an apoptosis detection result that demonstrated a low number of apoptotic cells throughout the ADSC spheroid (Supplementary data, Fig. S3D). The dissociated spheroids were lysed by freeze-thawing for 3 cycles to extract the soluble intracellular components in parallel with the generation of ADSC monolayer extract. Protein yield gained from ADSC spheroid extract was lower than ADSC monolayer extract, however, there was no statistically significant difference (Fig. 1D).

Alteration of MSC characteristics after priming with spheroid formation was assessed. The results revealed a decreased proportion of CD90, CD73, and CD105 positive cells in ADSC spheroids compared to ADSC monolayer (Fig. 1E). This is possibly due to the reduction of cell size when cultured as a spheroid. However, both ADSCs were negative for CD34 and CD45, a hematopoietic marker (Supplementary data, Table S4). Thus, priming ADSCs by spheroid culture modified the immunophenotype of ADSCs. Replating dissociated ADSC spheroids could adhere to polystyrene tissue culture vessels and displayed a fibroblast-like morphology (Fig. 1F). Like ADSC cultured in monolayer (Supplementary data, Fig. S4), ADSC spheroids retain a potent multilineage differentiation. The results showed that ADSC spheroids could differentiate toward adipocyte and osteoblast as assessed by positive staining for Oil Red O and Alizarin Red S, respectively (Fig. 1G–H).

### Preparation of the optimal condition for ADSC spheroid extracts

**Figure 2:**
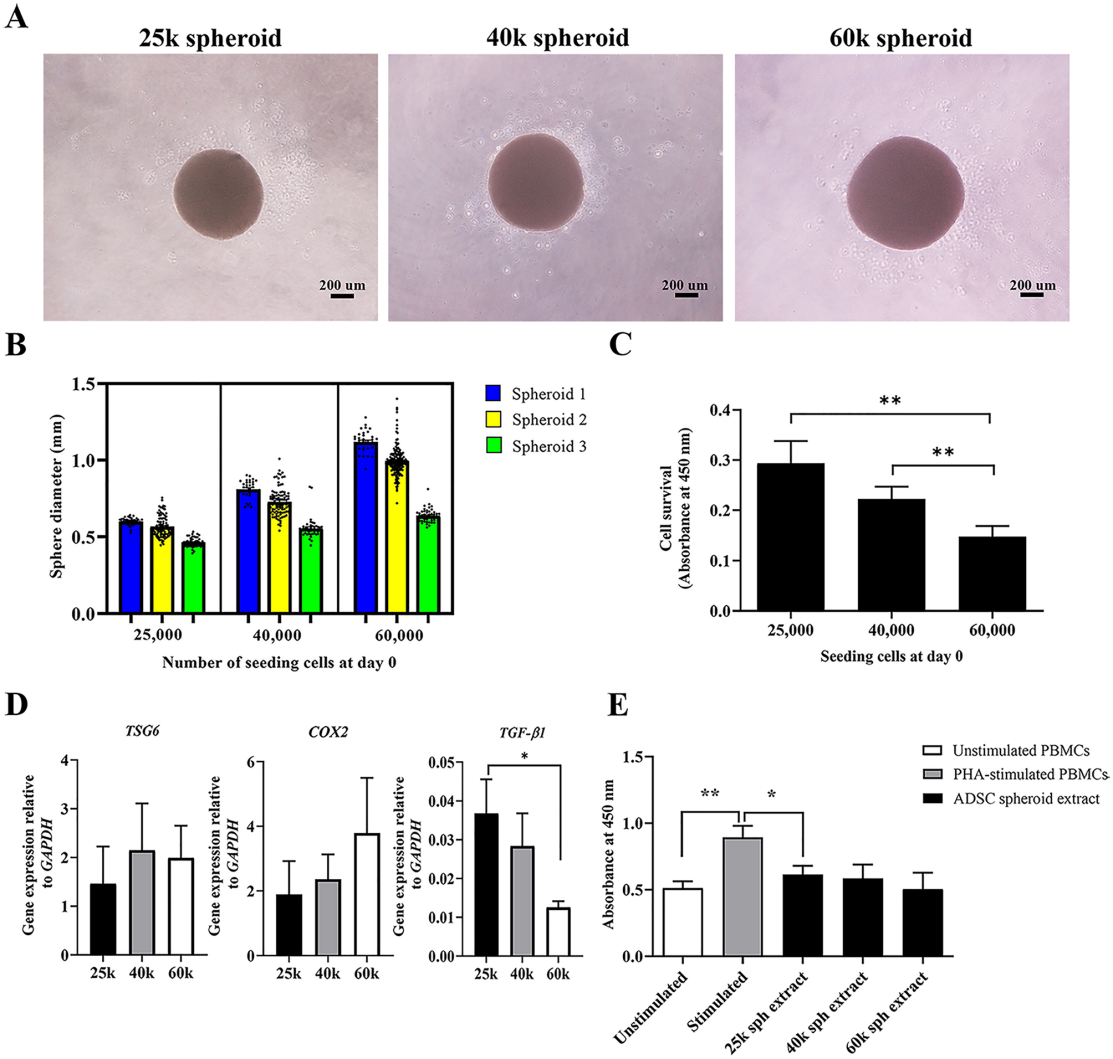
The effects of spheroid size distinction. (**A**) Morphology of spheroids from starting cells at 25,000 cells/spheroid (25 k), 40,000 cells/spheroid (40 k), and 60,000 cells/spheroid (60 k), respectively. The scale bar is 200 μm. (**B**) Size of spheroids generated from 3 ADSC donors cultured in the low-binding plate for 6 days. The diameter was measured by NIH Image J software (*n* = 35–96 for each ADSC donor). (**C**) Viable cells of dissociated 25 k, 40 k, and 60 k spheroid at day 6 of culture were determined by cell counting kit assay (*n* = 5). (**D**) The transcripts levels of *TSG-6*, *TGF-β1*, and *COX-2* in 25 k, 40 k, and 60 k spheroid. (**E**, **F**) PHA-stimulated PBMCs were treated with either 100 μg/ml intracellular extract-derived from 25 k, 40 k, and 60 k spheroid, respectively. PHA-stimulated PBMCs alone served as stimulated control. After 72 h-incubation, the numbers of lymphocytes were assessed by cell counting kit assay (*n* = 4). All data presents as mean ± SEM. *P < 0.05; **P < 0.01 versus controls; Mann–Whitney U test.

To identify the optimal condition of spheroid formation, the preparation of spheroid was performed by different cell seeding numbers varying from 25,000 to 60,000 cells. Most of the cell aggregations displayed spherical-shaped morphology (Fig. 2A). Spheroid diameter analysis revealed that the spheroid size depended on the number of seeding cells. The spheroids generated from 25,000 (25 k) ADSCs/spheroid offered a uniform spheroid size compared to the others (Fig. 2B). As expected, cell viability of spheroids significantly decreased in larger spheres (40,000 (40 k) and 60,000 (60 k) ADSCs/spheroid) (Fig. 2C). In addition, alterations in gene expression levels of the selected anti-inflammatory genes in individual spheroid size were investigated. The results showed that the transcripts levels of *TGF-β1*, an immunosuppressive factor were significantly higher expression in the spheroids of 25 k ADSCs than in 60 k ADSCs, whereas the expression of *TSG-6* and *COX-2* levels were not different (Fig. 2D).

To determine if the changes in spheroid density impact immunosuppressive ability, the effects of intracellular extracts derived from different spheroid sizes on lymphocyte proliferation were investigated. The results showed that all ADSC spheroid sizes could inhibit activated lymphocyte proliferation, but cellular extract derived from 25 k ADSC spheroid significantly decreased the number of activated lymphocytes. Increasing spheroid size did not alter the inhibition efficiency (Fig. 2E). Taken together, seeding 25 k ADSCs is suitable for spheroid formation which gains high expression of *TGF-β1* mRNAs, an immunomodulatory-associated gene. Moreover, intracellular extract derived from the spheroids of 25 k ADSCs efficiently suppresses lymphocyte activation.

### The effect of ADSC spheroid extracts on lymphocyte proliferation

**Figure 3:**
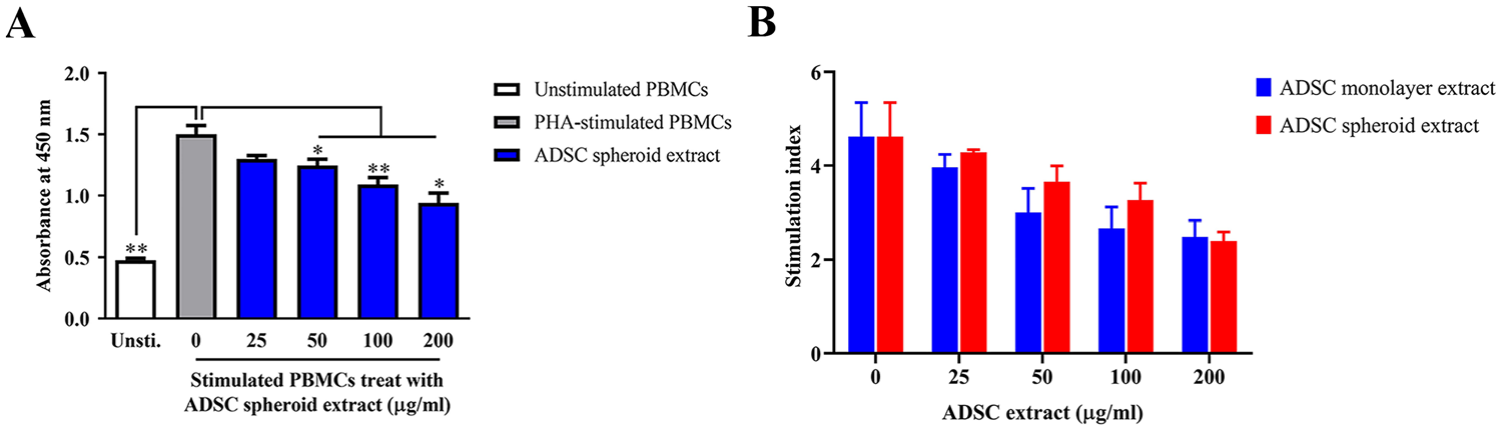
Effects of ADSC spheroid extract and ADSC monolayer extract on lymphocyte proliferation. (**A**) PHA-activated PBMCs were cultured in various spheroid extract concentrations (0, 25, 50, 100, and 200 μg/ml) for 72 h. Lymphocyte proliferation was assessed by cell counting assay (*n* = 4). (**B**) Comparison stimulation index of PBMCs of ADSC monolayer extract and ADSC spheroid extract on suppression of lymphocyte proliferation (*n* = 3). All data is presented as mean ± SEM. *P < 0.05; **P < 0.01 versus control which were tested by Mann–Whitney U test.

To investigate the immunomodulatory capacity of ADSC spheroid extract, PHA-activated lymphocytes were exposed to various concentrations of spheroid extracts. The numbers of viable lymphocytes were assessed after exposure with 25, 50, 100, and 200 μg/ml ADSC extracts for 72 h. PBMCs stimulated by PHA alone served as the control. Activation of lymphocytes with PHA could increase cell proliferation about 3 folds of unstimulated PBMCs. In contrast, activated lymphocytes were gradually reduced when treated with 50 μg/ml ADSC spheroid extract, 100 μg/ml ADSC spheroid extract, and 200 μg/ml ADSC spheroid extracts compared to the control (Fig. 3A). The results indicated that spheroid extracts dose-dependently suppressed activated T lymphocyte proliferation. The inhibition efficiency between ADSC spheroids extract and ADSC monolayer extract was compared to determine whether priming ADSCs by spheroid cultures enhances immunosuppression capacity. The results revealed that both ADSC spheroid extracts and monolayer extracts equally inhibit activated T cell proliferation (Fig. 3B). Overall, the results indicate that intracellular extract derived from both ADSC spheroid and ADSC monolayer efficiently mediated suppression of activated T lymphocyte proliferation.

### ADSC spheroid extracts mediate the T regulatory cell expansion

**Figure 4:**
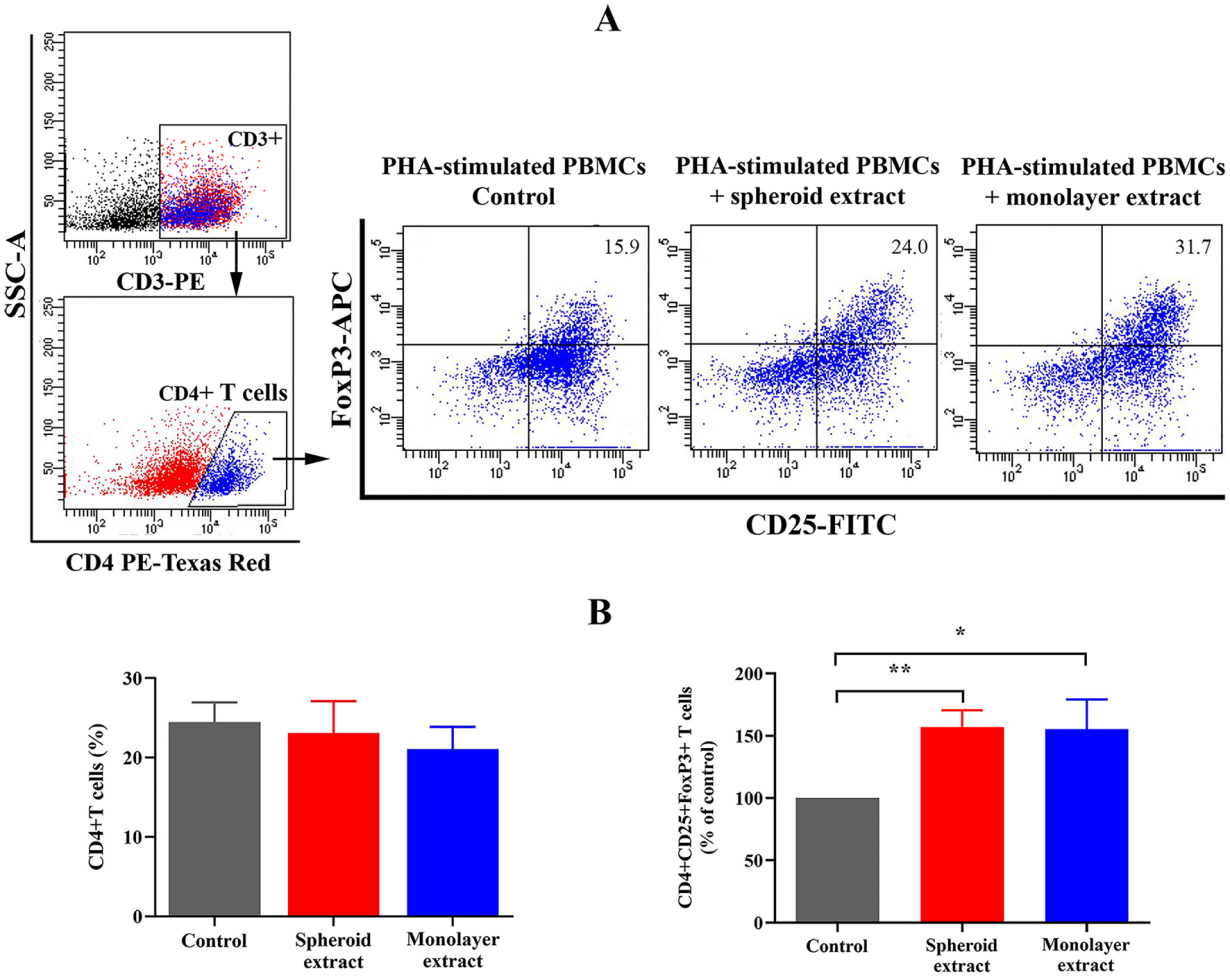
The effects of ADSC spheroid extract and ADSC monolayer extract on T regulatory cells. (**A**) PHA-stimulated PBMCs were treated with either 100 μg/ml ADSC spheroid extract or 100 μg/ml ADSC monolayer extract for 72 h. Cells gated on CD3 and CD4 are shown as scatter plots which represent one of the five independent experiments. T regulatory (T_reg_) cell population was defined by CD4 ^+^ CD25 ^+^ FoxP3 ^+^ T cells. The percentage of Treg cells from one representative experiment was presented on the top right quadrant of each plot. (**B**) Percentage of CD4^+^ T cells and CD4^+^CD25^+^FoxP3^+^ T_reg_ cells (*n* = 5). All data presented as mean ± SEM. *P < 0.05; **P < 0.01 versus controls which were tested by one-way ANOVA followed by the Kruskal–Wallis test.

T regulatory cell analysis was performed to better understand the immunomodulatory effects of ADSC extracts. PHA-activated lymphocytes were treated with either ADSC spheroid extracts or ADSC monolayer extracts for 72 h. PBMCs stimulated by PHA alone served as the control. T regulatory cell subpopulation was assessed based on the CD4^+^CD25^+^FoxP3^+^ T cell population (Fig. 4A). The results showed that no difference was found in the percentage of CD4^ +^ T cells when cultured lymphocytes in the presence of either ADSC spheroid extracts or ADSC monolayer extracts compared with control (Fig. 4B). Interestingly, both spheroid extracts and monolayer extracts significantly increased the percentage of regulatory T cells as defined by CD4 ^+^ CD25 ^+^ FoxP3 ^+^ T cells population (Fig. 4A,B). In ADSC spheroid extract treatment, the percentage of CD4 ^+^ CD25 ^+^ FoxP3 ^+^ T cells was 1.59-fold higher than in PHA-activated T lymphocyte alone. Meanwhile, the percentage of regulatory T cells was 1.61-fold increased upon ADSC monolayer extract exposure. The results indicate that intracellular extracts from both ADSC spheroid and ADSC monolayer contain bioactive molecules playing roles in enhancing T regulatory cell expansion.

### Cultures ADSC as spheroids up-regulate TGF-β1, COX-2, and TSG-6

**Figure 5:**
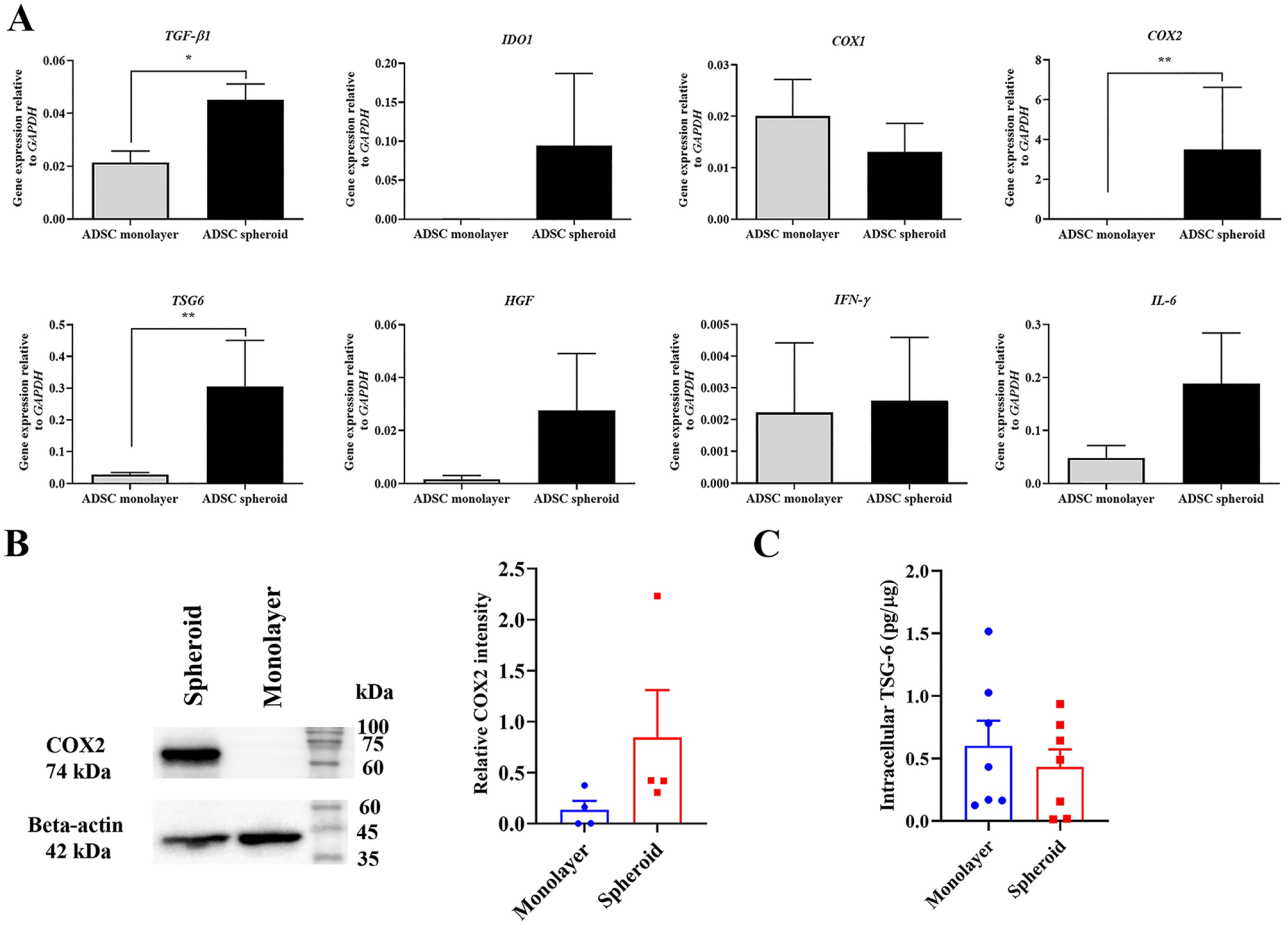
The expression of immunomodulatory molecules. (**A**) The transcriptional levels of immunomodulatory molecule-associated genes (*TGF-β1, IDO-1, COX-1, COX-2, TSG-6*, and *HGF*) and inflammatory-associated genes (*IFN-γ* and *IL-6*) were examined after cultured ADSC as spheroid and monolayer for 6 days. (**B**) Western blotting analysis of COX-2 expression in ADSC spheroid extract and ADSC monolayer extract. The blot image represents one of the four independent samples. Full-length blots are presented in Supplementary Fig. S5. The band intensity of COX-2 relative to β-actin, the internal control was evaluated (*n* = 4). (**C**) Intracellular TSG-6 normalized to total cellular protein of ADSC spheroid extracts and ADSC monolayer extracts from the same donors (*n* = 7). All data is presented as mean ± SEM. *P < 0.05; **P < 0.01 versus ADSC monolayer; Mann–Whitney U test.

To determine the changes in gene expression profile after ADSC spheroid cultures, certain immunomodulatory molecule-associated genes were analyzed compared with the conventional monolayer culture. Analysis of the gene expression profile revealed that ADSC spheroid significantly up-regulated the expression of genes involved in immunomodulation and anti-inflammation mediators including *TGF-β1*, *COX-2*, and *TSG-6* (Fig. 5A). The *TGF-β1* expression increased over twofold compared to ADSC monolayer, while *TSG-6* increases over tenfold. The expression of *COX-2,* but not *COX-1* raised over 300-fold compared to ADSC monolayer. However, the transcriptional levels of *IDO-1*, *HGF*, *IFN-γ*, and *IL-6* were not significantly different. According to the transcriptional profile results, the protein levels of COX-2 and TSG-6 in ADSC spheroid and monolayer extracts were further investigated. Western blotting demonstrated the expression of COX-2 protein in intracellular extracts from both ADSC monolayer and ADSC spheroid. The ADSC spheroid extract contained a higher COX-2 protein level than the ADSC monolayer extract that was prepared from the same donor (Fig. 5B). Although ADSC spheroid increased the transcriptional levels, the levels of intracellular TSG-6 protein in ADSC spheroid extract and ADSC spheroid extract were not different (Fig. 5C). Taken together, ADSC spheroid extract has the potential to establish an immunosuppression environment by generation of certain anti-inflammatory and immunomodulatory molecules.

### Cyclooxygenase-2 plays a role in supporting T regulatory cell expansion

**Figure 6:**
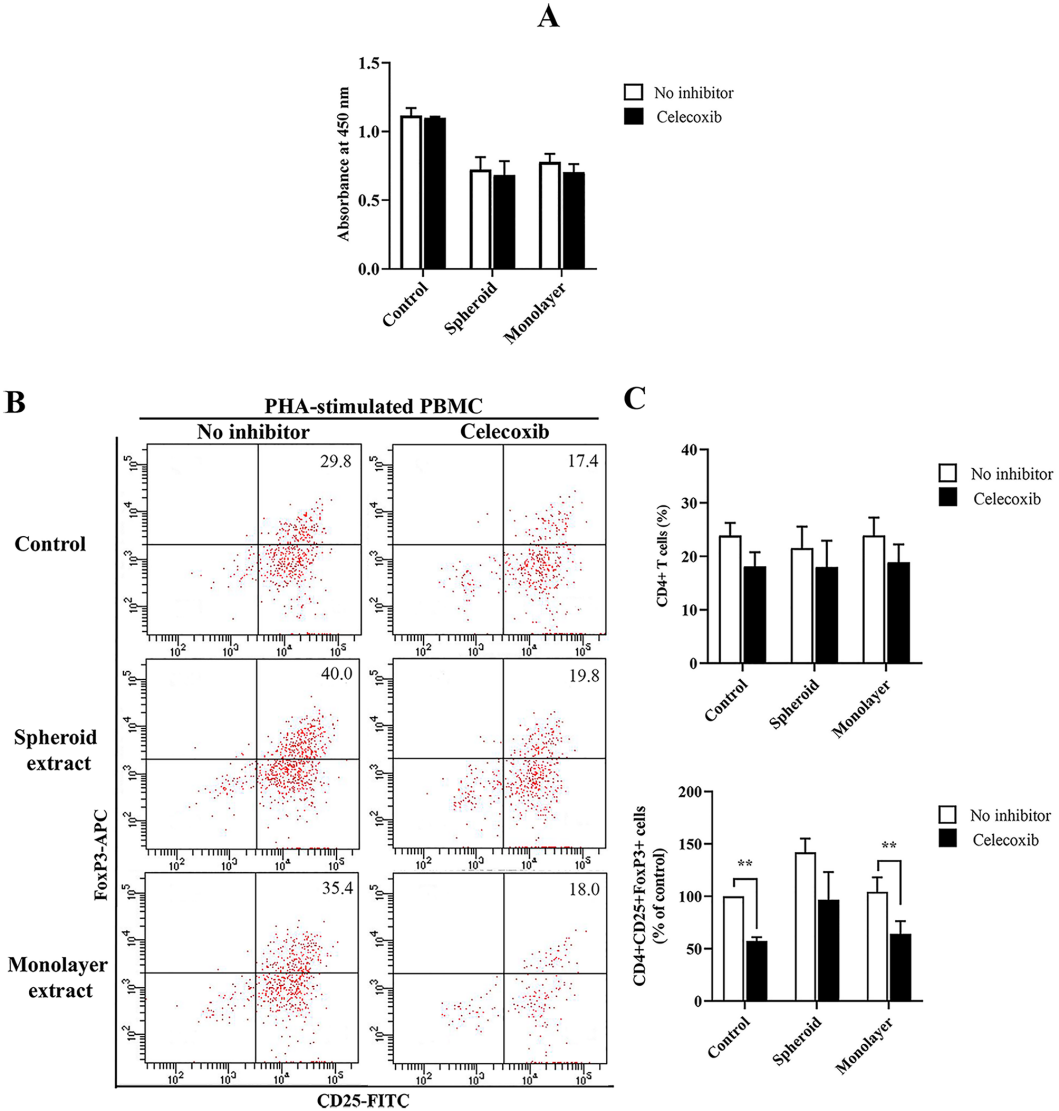
Roles of selective COX-2 inhibitor on immunomodulatory effects of ADSC extract. PHA-stimulated PBMCs were treated with either 100 μg/ml ADSC spheroid extract or 100 μg/ml ADSC monolayer extract together with 10 μM celecoxib, a selective COX-2 inhibitor for 72 h. The PHA-stimulated PBMCs cultured without ADSC extract treatment serve as control. (**A**) Roles of COX-2 inhibitor on lymphocyte proliferation capacity were investigated by CCK8 assay (*n* = 3). (**B**) Scatter plots represent one of the four independent experiments showing the T regulatory (T_reg_) cell population with gating of CD25 ^+^ FoxP3 ^+^ cells from CD3 ^+^ CD4 ^+^ T cells fractions. (**C**) The percentage of CD4 ^+^ T cells and CD4^+^CD25^+^FoxP3^+^ T cells are presented as the percentage of PHA-stimulated PBMCs culture alone (*n* = 4). All data is presented as mean ± SEM. *P < 0.05; **P < 0.01 versus no inhibitor; Pair *t*-test.

We clarify whether COX-2, which is overexpressed in ADSC spheroid represents a key molecule in modulating immune cell functions. Celecoxib concentration was optimized before performing the inhibition assay. Celecoxib, a selective COX-2 inhibitor was added in PHA-stimulated PBMCs in the presence or absence of ADSC extracts and cultured for 72 h. The result showed that the proliferation capacity of activated PBMCs did not interfere with celecoxib addition. In celecoxib treated together with ADSC extract, COX-2 inhibitor could not recover the effect of ADSC spheroid extract and ADSC monolayer extract on suppression of activated T cell proliferation (Fig. 6A). The observation indicates that other immunomodulatory mediators may play roles in suppressing T cell activation. However, the effect of ADSC extract on the induction of the T regulatory population was abrogated by COX-2 inhibition (Fig. 6B,C). The percentage of CD4 ^+^ T cells was not affected by COX-2 inhibition when compared to untreated control (Fig. 6C). By addition of celecoxib, the proportion of CD4 ^+^ CD25 ^+^ FoxP3 ^+^ T regulatory cells significantly decreased in control (57.30 ± 3.71% of untreated control) and ADSC monolayer extract treated group (64.12 ± 12.11% of untreated control). Inhibition of COX-2 in the presence of ADSC spheroid extracts reduced the percentage of T regulatory cells from 141.80 ± 13.29% to 96.82 ± 26.36% of untreated control, however, there was no statistical difference. This finding suggests that COX-2 is one of the immunomodulatory molecules that mediate T regulatory cell induction.

### Effect of ADSC extracts on in vitro macrophage immunomodulation

**Figure 7:**
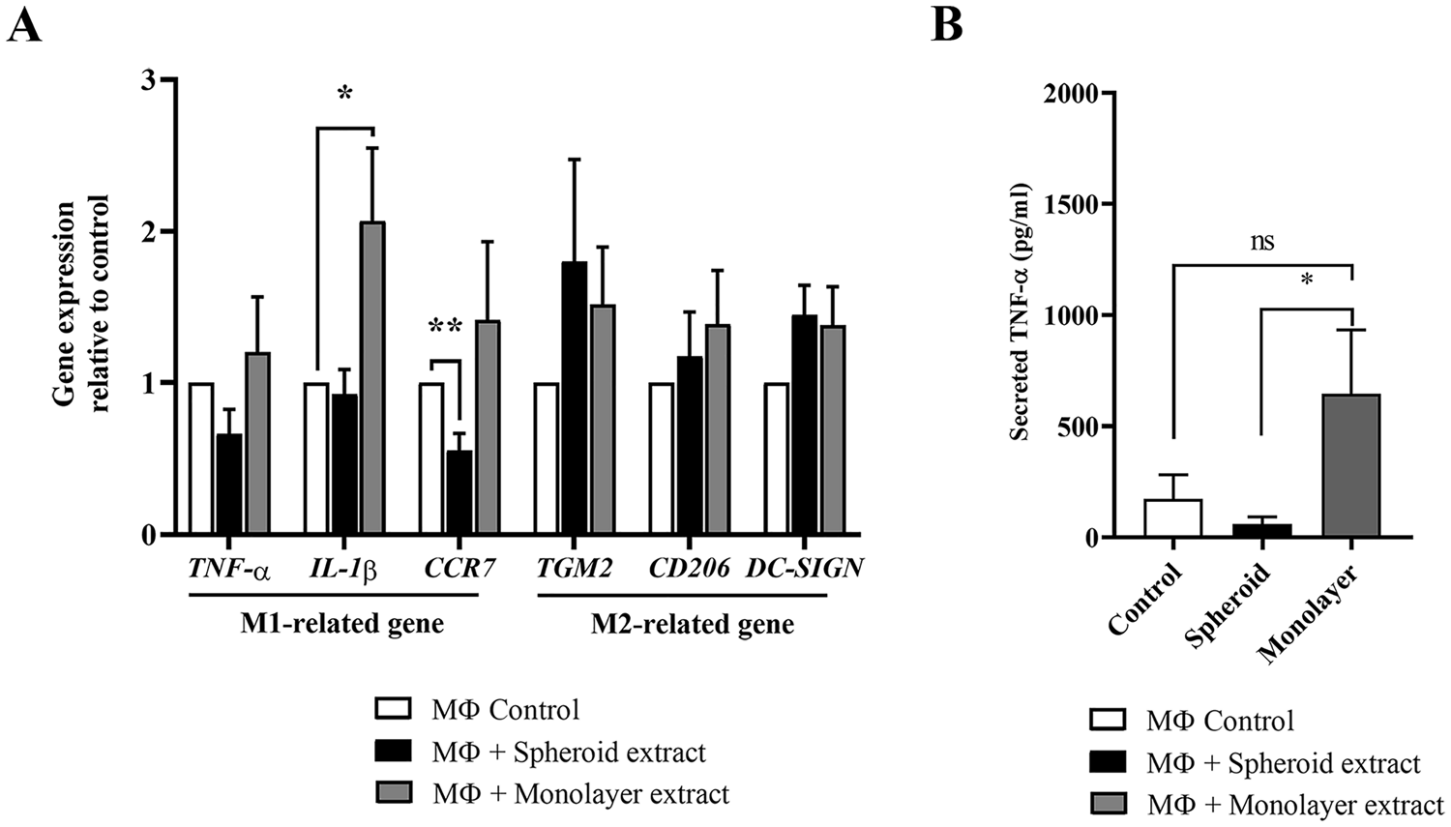
In vitro macrophage assay. The roles of ADSC spheroid extract and ADSC monolayer extract in mediating the phenotypic shift of macrophage M1 toward M2 macrophage. (**A**) The transcripts levels of M1-related genes (*TNF-α*, *IL-1β*, and *CCR7*) and M2-related genes (*TGM2*, *CD206*, and *DC-SIGN*) were determined after treatment with ADSC extracts for 24 h (*n* = 4). (**B**) The concentration of secreted TNF-α produced by LPS-treated macrophage after exposure to ADSC spheroid extracts and ADSC monolayer extracts for 24 h (*n* = 5). All data is presented as mean ± SEM. *NS* Not significant, *P < 0.05; **P < 0.01 versus macrophage control which were tested by Mann–Whitney U test.

Together with high expression of anti-inflammatory mediators, ADSC spheroid extract would be more effective in the modulation of macrophage functions. Thus, the additional role of ADSC spheroid extracts in regulating inflammation was determined using an in vitro macrophage assay. We first investigated the influence of ADSC spheroid extract on macrophage survival. PMA-differentiated THP-1 cells were cultured in the presence of various ADSC spheroid extract concentrations (25, 50, 100, and 200 μg/ml) for 24 h, 48 h, and 72 h, respectively. The results revealed that ADSC spheroid extract had little effect on macrophage survival, even with increased treatment concentrations (Supplementary data, Fig. S6). To examine the roles of ADSC extract in the modulation of macrophage phenotype, LPS-induced PMA-differentiated THP-1 cells were exposed with either ADSC spheroid extract or ADSC monolayer extract while LPS-induced PMA-differentiated THP-1 cells cultured alone served as macrophage (MΦ) control. After 24-h incubation, the expression of M1 and M2 macrophage-related genes were assessed. The results showed that spheroid extract exposure downregulated the M1-related genes, especially *CCR7* compared with MΦ control. On the contrary, the addition of monolayer extract upregulated the expression of *IL-1β* to twofold compared with MΦ control (Fig. 7A). The transcripts levels of M2-related genes including *TGM2*, *CD206*, and *DC-SIGN* in both ADSC spheroid extract and ADSC monolayer extract treatment found no significant differences. Interestingly, the levels of secreted TNF-α from LPS-stimulated macrophages were reduced in ADSC spheroid extract treatment (60.22 ± 32.23 pg/ml) but increased in monolayer extract treatment (645.3 ± 287.9 pg/ml) when compared with control (173.9 ± 108.8 pg/ml) (Fig. 7B). The finding indicated that spheroid extract obtained superior anti-inflammatory efficacies, whereas monolayer extract tended to induce an inflammatory phenotype in macrophages.

## Discussion

Immunomodulatory capacities of mesenchymal stem cells have been reported in both in vitro and in vivo studies^12,23^. These beneficial effects are mediated in part by the secretion of various immunosuppressive mediators. Herein, we aimed at the generation of cell-free MSC products with high efficacy of immunomodulation and anti-inflammation by simply pre-priming MSCs through spheroid culturing. Priming strategies include proinflammatory cytokines exposure, hypoxia, and three-dimensional cultures showing increased secretion of bioactive molecules that play a role in anti-fibrosis, angiogenesis, cellular regeneration, and immunoregulation^24,25^. Thus, preconditioning MSCs is essential for enhancing the MSC potency. Spheroid cultures closely recapitulate in vivo MSCs. In agreement with a previous study, our finding demonstrated that ADSC spheroid alters the expression of MSC surface markers especially, CD105 but maintains multilineage differentiation potential^26^. A decline in CD105 expression probably results from changes in cell size and alteration of adhesion molecule expression^27^. Based on the flow cytometry analysis, ADSCs from spheroids were smaller than ADSCs from monolayers. These morphological changes could alter the levels of cell surface marker expression. The morphological changes may be one of the factors that impact the immunomodulatory efficiency of ADSCs. This agrees with the prior studies, which demonstrated that alteration of IFN-γ-primed MSC morphology correlate with the immunosuppressive efficiency of MSCs^28^. CD105 is a component of TGF-βR type I and II, which is presented in both extracellular and cytoplasmic domains. The interaction between CD105 and TGβRI or TβRII plays a role in modulating TGF-β signaling. A Change in CD105 expression may regulate the cellular responses to TGF-β1. A previous study demonstrated that the expression of CD105 was affected by cell culture procedures. Conversely, reducing CD105-positive cells did not affect MSC differentiation capacities or immunomodulatory properties^29^. Thus, further investigation on the presence of extracellular and cytoplasmic endoglin in ADSC extract would clarify whether CD105 alteration in ADSC spheroid impact the immunosuppressive capacities of the intracellular extract.

Moreover, the generation of optimal spheroid size should be considered for the preparation of ADSC spheroid extract that obtains immunomodulation properties. Comparable to a previous study, initial cell seeding of 25,000 cells for a single spheroid is sufficient for forming an ADSC spheroid with potent immunosuppression capacity^17^. However, the presence of an apoptotic and necrotic core in the spheroid might impact the functional properties of the cellular extract. Thus, a cell viability assay is necessary to assess the quality of ADSC spheroid prior to the intracellular extraction. Herein, the cell viability of 25,000 ADSCs/spheroid was investigated using a trypan blue assay and an Annexin V/PI apoptosis assay. In line with previous studies, the viable cells throughout the spheroids were more than 90%, while less than 5% were apoptotic cells. Prior studies demonstrated that the largest spheroids (60,000 cells/spheroid) increased caspase activity, which indicated higher cellular apoptosis than the smaller spheroids (15,000–30,000 cells/spheroid)^30^. The increase in spheroid size led to reduced cell viability, which possibly resulted from the difficulty of nutrient penetration and the hypoxic environment in the core of spheroid^26^.

Cellular lysates are involved in biological activities, such as cell reprogramming and tissue repair, therefore they contain multiple bioactive molecules^31,32^. Due to the efficacy of cellular extract, it raises the possibility of using intracellular components as therapeutic cell-free products. In the present study, we first reported that an intracellular extract derived from ADSC spheroids could suppress activated T cell proliferation in a dose-dependent manner. Moreover, ADSC spheroid extracts enhanced the expansion of regulatory T cells. Surprisingly, both ADSC spheroid extract and ADSC monolayer extract showed comparable efficiency in suppressing T cell activation and inducing of T regulatory cells. Thus, intracellular ADSC spheroid and ADSC monolayer extracts might share common immunomodulatory factors that are sufficient for the regulation of T lymphocyte functions. Proteomic profiling of the MSC secretome revealed bioactive molecules that play a major role in biological processes including cell growth and maintenance, cellular communication, cell survival, and angiogenesis^2,3^. Previous studies demonstrated that MSC derived from adipose tissues showed a high potency of immunosuppression over bone marrow-derived MSC by secretion abundant amounts of IL-6 and TGF-β1^33^. Our findings highlight the fact that intracellular ADSC extracts possess immunosuppressive capacities.

Alterations in transcriptome profiles between MSC spheroids and MSC monolayer have been identified by prior studies using microarray assay^26^. This transcriptome analysis demonstrated a change in gene expression profiles of MSC spheroid, especially the increase in genes related to wound healing and inflammation response compared with MSC monolayer. Nevertheless, the current understanding of the immunoregulatory role of cellular extract is limited. In this study, comparative transcriptional profiles revealed that the ADSC spheroid upregulates the transcriptional levels of *COX-2*, *TGF-β1*, and *TSG-6*. In addition, the COX-2 proteins were highly present in ADSC spheroid extract over ADSC monolayer extract. In line with previous studies, priming MSCs through spheroid formation enhances the production of a plethora of immunomodulatory mediators, especially STC, IDO, HGF, PGE2, TSG-6, and TGF-β^26^. In addition, preconditioning MSC spheroid with proinflammatory cytokines such as IL-1 and IFN-γ further promoted the expression of immune suppressive profiles and increased secretion of anti-inflammatory factors^16,34^. Thus, the secretome produced from MSCs and its immunomodulation properties are greatly influenced by the microenvironment for cell culture and the morphological changes^17,28,35^. The underlying mechanism by which spheroid formation exhibits enhanced functions and increased bioactive molecules expression remains obscure. It is possible that spheroid formation creates a hypoxic core, promotes cell–cell interactions, and induces cellular stress. These may affect cell behavior, cell functions, and the production of bioactive molecules. In addition, the enhanced functions of spheroid may be mediated via an alteration of metabolic activity and activation of caspase 3/7 activity rather than a hypoxic core^30^. During spheroid formation, MSCs self-activated IL-1 signaling and Notch signaling, which are responsible for inducing TSG-6, STC1, and PGE2 production^36^. Taken together, culturing ADSCs as spheroids altered cell morphology, surface markers, gene expression profiles, and the production of immunomodulatory mediators.

Cyclooxygenase-2 (COX-2) is an enzyme that catalyzes arachidonic acid, resulting in PGE2 production. MSCs secrete PGE2 in response to inflammatory signals and spheroid formation, which mediate immunomodulatory effects in immunological diseases and tissue regeneration models^15,37^. Interestingly, selective inhibition of COX-2 abrogates the effects of ADSC spheroid extracts and ADSC monolayer extracts on inducing T regulatory cell expansion. In comparison with a prior study that reported COX-2 is important for the suppression of T cell proliferation^38^, our results indicated that COX-2 inhibition by celecoxib was incapable of attenuating the immunosuppression efficiency of MSCs on activated T cell proliferation. Therefore, COX-2 is one of the key factors that partially mediates the immunomodulation effects of ADSC extracts. This agrees with the prior studies, which showed that MSCs could suppress T cell activities through the COX-2/PGE2 pathway. PGE2 inhibits IL-2 production, leading to the suppression of lymphocyte proliferation and activation^39,40^. In this study, selective COX-2 inhibition did not entirely block the COX-1/PGE-2 pathway or the residual PGE-2. Thus, the difference in immunosuppressive effects of ADSC spheroid extract within COX-2 inhibition could be related to the levels of PGE-2. Apart from PGE-2, there are other undefined factors in the intracellular compositions that might suppress the activated T cell proliferation such as IL-10, TGF-β1, and IDO^24,41^. The upregulation of *TGF-β1* and *TSG-6* transcripts in the ADSC spheroids implies the abundant presence of their proteins in the intracellular extract. TGF-β1 plays an essential role in regulating T cell activities such as activation, proliferation, and differentiation. TGF-β1 stimulates T regulatory cell expansion and induce T regulatory cell differentiation via upregulation of *FOXP3* gene^42^. Similarly, COX-2/PGE-2 could induce *FOXP3* expression and enhance T regulatory cell function^43^. A previous study demonstrated that PGE2 and TGF-β1 had a non-redundant role in the induction of T regulatory cells by MSCs^44^. Taken together, abundant immunomodulatory molecules, notably COX2, TGF-β1, and TSG-6 in ADSC spheroid extract, strongly support the immunosuppressive potential of the extract on T cell functions.

The release of soluble bioactive molecules such as IDO, NO, TSG-6, IL-10, VEGF, and PGE2 by MSCs has been shown to control inflammation and stimulate tissue regeneration through modulation of macrophage (MΦ) plasticity^45,46^. The interaction of MSCs and macrophages induced the shift of inflammatory (M1) macrophage polarization toward anti-inflammatory (M2) macrophage phenotype^47^. In the current study, the anti-inflammatory capacity of ADSC was enhanced even further when the intracellular extracts were produced from ADSC spheroids. The levels of inflammatory factors, such as the *CCR7* gene and the secreted TNF-α protein, were decreased in LPS-induced macrophage exposed to ADSC spheroid extracts, while M2-associated genes slightly increased. On the contrary, unprimed ADSC extract treatment triggered an inflammatory M1 phenotype as LPS-induced macrophages increased TNF-α production. The ADSC spheroid extracts attenuate the inflammatory capacity of LPS-induced macrophages. From these findings, it is clear that ADSC spheroid extracts were more effective in regulating macrophage inflammation when compared to ADSC monolayer extracts. Based on previous evidence, the PGE2-presented MSC-spheroid secretome plays a major role in the alteration of LPS-stimulated macrophages toward the anti-inflammatory M2 phenotype. Inhibition of PGE2 abrogated the anti-inflammatory capacity of MSCs^15,36^. PGE2 binds EP2 and EP4 receptors on macrophages and activates the downstream signaling pathway. This activation increases *C/EBP-β* expression, which then promotes M2 macrophage polarization by enhancing M2 markers ARG1 and CD206 expression and induces IL-10 production^45^. PGE2 secreted by MSC through the COX-2 pathway modulates macrophage metabolism, leading to the shifting M1 to M2 macrophages^48^. In addition, upregulation of the *TSG-6* gene was found in ADSC spheroid. A prior study showed that TSG-6 could drive the anti-inflammatory phenotype of macrophages^49^. Our interpretation was that priming ADSC with spheroid formation improved its anti-inflammatory abilities. The COX-2 and TSG-6 produced by ADSC spheroid may be responsible for mediating anti-inflammatory ability.

The current study demonstrated that ADSC spheroid extract gains an in vitro immunomodulatory property. However, it is unclear whether the capacity is maintained following administration in an in vivo model. Further testing of the ADSC spheroid extract’s efficacy in an in vivo model of immunological diseases is warranted. Additionally, it is important to identify the absolute amount of candidate proteins, optimize the effective dosage of cellular extract for administration in vivo*,* and determine the duration of activity. Furthermore, the immunogenicity of the cellular extract should be determined when transferring an in vitro study to an in vivo model and clinical applications to ensure the safety of the cellular extract.

## Conclusion

To conclude, priming ADSC via spheroid cultures enhances the immunoregulatory potential of ADSCs by raising the production of immunomodulatory mediators in the intracellular composition. Thus, ADSC spheroid extract may potentially offer promising cell-free MSC therapeutic products for immunological diseases. However, the current study partially identified key immunomodulatory factors. Proteomic profiling of ADSC spheroid extracts and in vivo study should be further conducted to clarify the composition of the extract and its mechanism of action. In addition, high-throughput technology is necessary for the massive generation of ADSC spheroids and intracellular components for therapeutic purposes.

### Supplementary Information

Supplementary Information.

## Data Availability

The datasets used and/or analyzed during the current study are available from the corresponding author on reasonable request.

## Abbreviations

ARG1: Arginase 1
COX-2: Cyclooxygenase 2
IDO: Indoleamine 2,3-dioxygenase
IFN-γ: Interferon-γ
IL-1β: Interleukin-1β
IL-6: Interleukin-6
IL-10: Interleukin-10
NO: Nitric oxide
PGE2: Prostaglandin E2
STC-1: Stanniocalcin-1
TNF-α: Tumor necrosis factor alpha
TGF-β1: Transforming growth factor beta-1
TSG-6: Tumor necrosis factor-inducible gene 6
