# Supplementary material for: Enhanced potent immunosuppression of intracellular adipose tissue-derived stem cell extract by priming with three-dimensional spheroid formation
Source: Sci Rep. 2024 Apr 20;14:9084. doi: 10.1038/s41598-024-59910-x (PMC11032398; doi:10.1038/s41598-024-59910-x)
Supplement: Supplementary file 1 — Supplementary Information. [file 41598_2024_59910_MOESM1_ESM.pdf]

Enhanced potent immunosuppression of intracellular adipose tissue-derived stem cell extract by priming with three-dimensional spheroid formation

Witchayapon Kamprom[1], Rattanawan Tangporncharoen[2], Nuttapoom Vongthaiwan[2], Patcharapa Tragoonlugkana[2], Jitrada Phetfong[2], Chatchai Pruksapong[3], and Aungkura Supokawej[2*]

Supplementary data

Table 1 List of antibodies for flow cytometry analysis

Target	Conjugation	Reference/ clone	isotype	Brand
CD73	PE/Cy7	AD2	Mouse IgG1κ	Biolegend
CD90	APC	REA897	Recombinant human IgG1	Miltenyi Biotec
CD105	PE	MEM-226	Mouse IgG2a	EXBIO
CD34	PE	8G12	Mouse BALB/c IgG1, κ	BD Bioscience
CD45	PerCP	5B1	Mouse IgG2aκ	Miltenyi Biotec
CD4	PE-Texas red	RPA-T4	Mouse IgG1κ	BD Bioscience
CD25	Viobright 515	REA570	Recombinant human IgG1	Miltenyi Biotec
FoxP3	Vio 667	REA944	Recombinant human IgG1	Miltenyi Biotec

Table 2 List of oligoes primers for quantitative real time PCR

Gene name	Forward primer	Reverse primer
TGF-β1	5'GGCCGGTAGTGAACCCGTTG3'	5'GGCCGGTAGTGAACCCGTTG3'
IDO-1	5'GTGGGCTTTGCTCTGCCAAA3'	5'GCGCTGTGACTTGTGGTCTG3'
IFN-γ	5'GTGGGCTTTGCTCTGCCAAA3'	5'GCGCTGTGACTTGTGGTCTG3'
IL-6	5'TTCGGTACATCCTCGACGGC3'	5'TCTGCCAGTGCCTCTTTGCT3'
COX-1	5'TTGCTGTTCCTGCTCCTGCT3'	5'CCCTGGTGCTGGCATGGATA3'
COX-2	5'TGCCTGATGATTGCCCGACT3'	5'TGAAAGCTGGCCCTCGCTTA3'
HGF	5'CACGAACACAGCTTTTTGCCTT3'	5'ACTCTCCCCATTGCAGGTCA3'
TSG-6	5'GCGGCCATCTCGCAACTTAC3'	5'TTGGGCCCTGGCTTCACAAT3'
IL-10	5'AAGACCCAGACATCAAGGCG3'	5'CAGGGAAGAAATCGATGACAGC3'
TNF-α	5'GCCTCTTCTCCTTCCTGATCG3'	5'GCTTGAGGGTTTGCTACAACA3'
IL-1β	5'GAGCTCGCCAGTGAAATGATG3'	5'TGGTCGGAGATTCGTAGCTG3'
CCR7	5'GGCTGGTCGTGTTGACCTAT3'	5'ACGTAGCGGTCAATGCTGAT3'
CD206	5'CAGACACGATCCGACCCTTC3'	5'GTCTCCGCTTCATGCCATTG3'
DC-SIGN	5'CCTACAGCTGCAGTCTTCCA3'	5'TACTGCTTGAAGCTGGGCAA3'
TGM2	5'GCCCACATCACCAACAACAC3'	5'GTAGAGGTCCCTCTCAGCCA3'
GAPDH	5'CAACTACATGGTTTACATGTTCCAA3'	5'CAGCCTTCTCCATGGTGGT3'

Table 3 Immunophenotype of ADSCs derived from adipose tissues of individual donor

**Cell surface markers (%)**	**ADSC #1**	**ADSC #2**	**ADSC #3**	**Total** (Mean±SEM)
MSC markers				
CD73	99.3	98.2	96.3	97.93±0.88
CD90	99.5	100	96.2	98.57±1.19
CD105	94	95.2	99.8	96.33±1.77
Hematopoietic markers				
CD34	1.9	0.6	3.8	2.10±0.93
CD45	0.2	1.2	0.3	0.57±0.32

Table 4 Expression levels of MSC surface markers in ADSC spheroid compared to ADSC monolayer

Cell surface markers	ADSC spheroids positive cells (%)	ADSC monolayer positive cells (%)
MSC markers		
CD73	87.8±2.6	99.3±0.2
CD90	74.5±13.0	95.4±2.9
CD105	6.5±4.4	94.4±2.9
Hematopoietic markers		
CD34	1.3±0.6	2.4±1.4
CD45	0.1±0.1	1.8±1.8

Data are presented as the mean±SEM ($n = 3$)

Supplementary figures

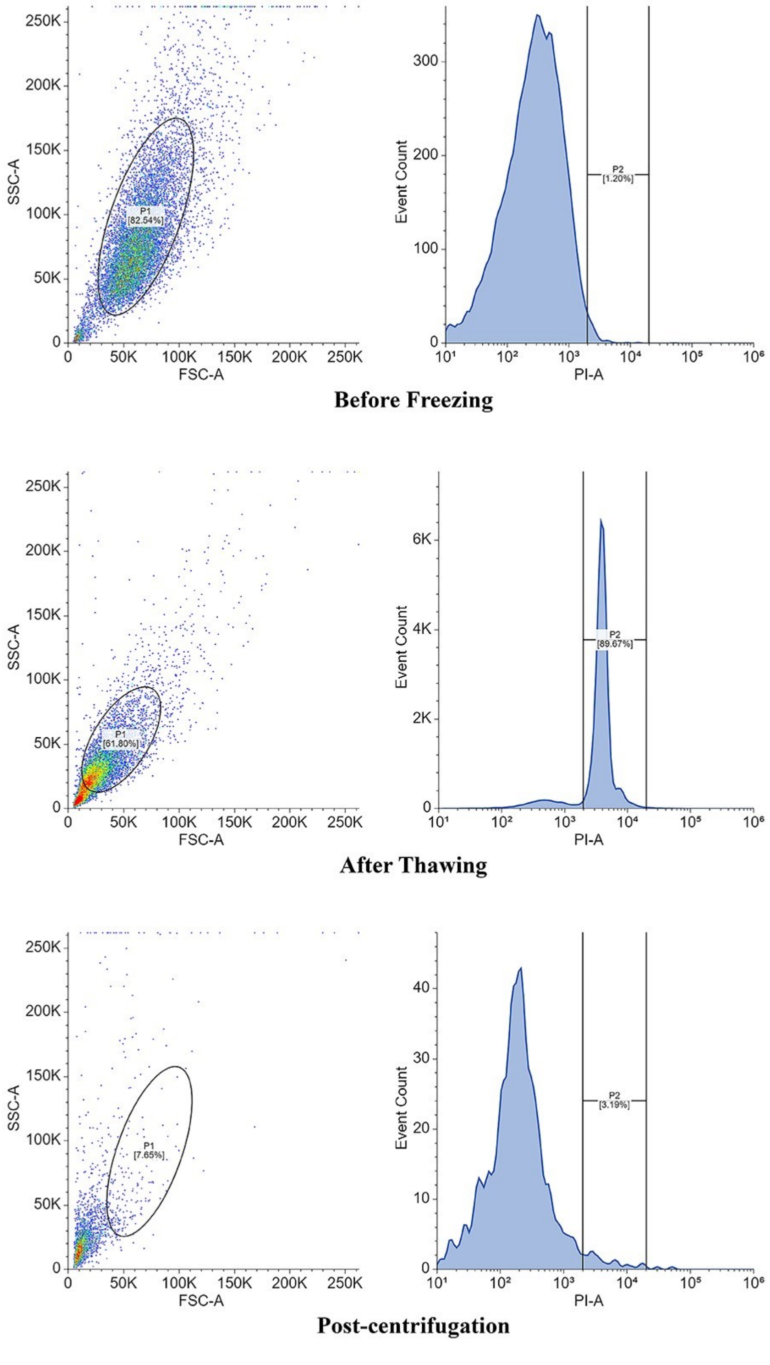

Figure S1. Cell debris and nuclear fragment investigation. The presence of cell debris and nuclear fragments in cell suspension was investigated at various steps of cellular extract preparation which consisted of the cells before freezing at -80°C, the cells after freeze-thawing for 3 cycles, and the cells after removing cell debris by centrifugation, respectively. The scatter plot indicated the percentage of cell population at each step of cellular extract preparation. Histogram presents the percentage of nuclear fragments (PI+).

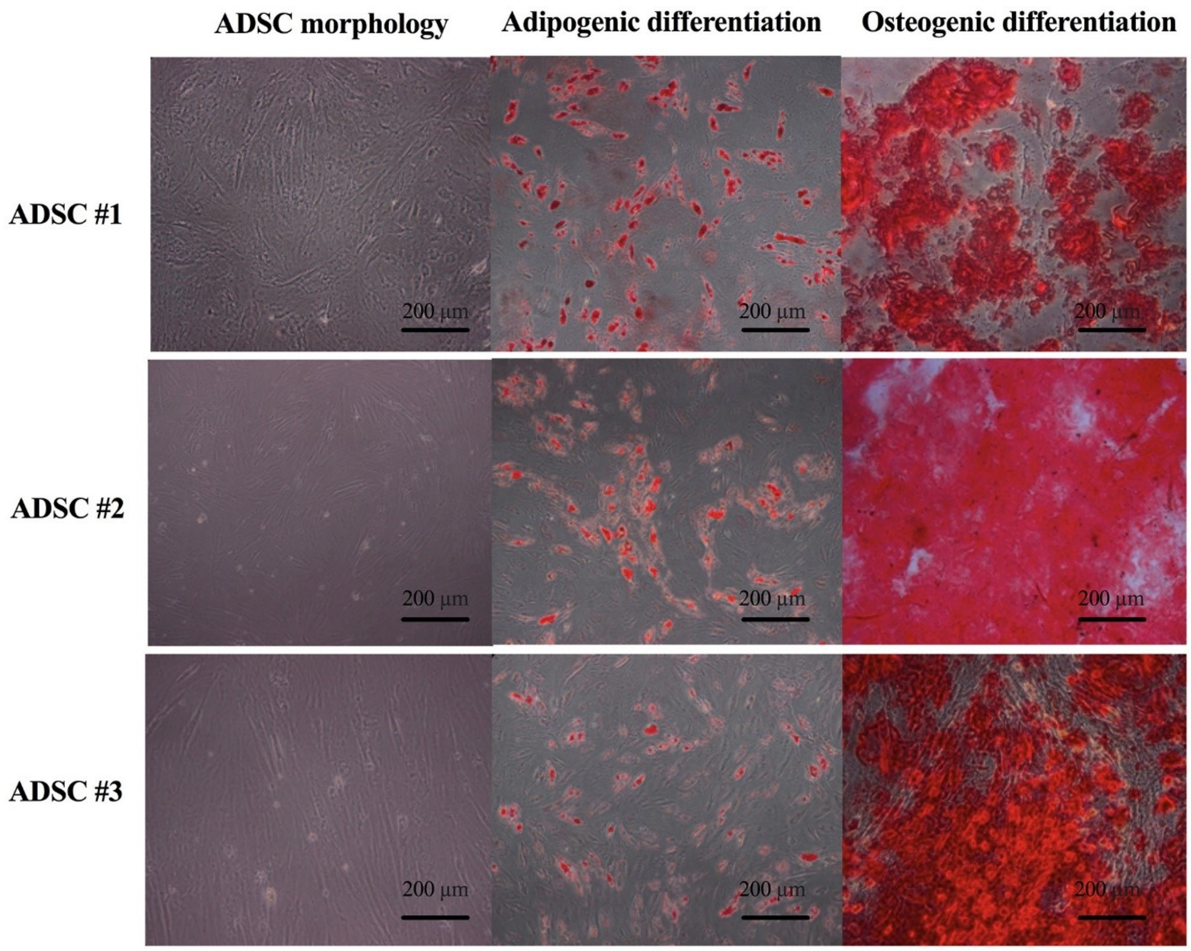

Figure S2. ADSC characteristics. The differentiation capacities of individual ADSC donors were investigated prior to spheroid formation. All ADSCs showed fibroblast-like morphology and could differentiate to adipocyte and osteoblast, respectively (*n*=3).

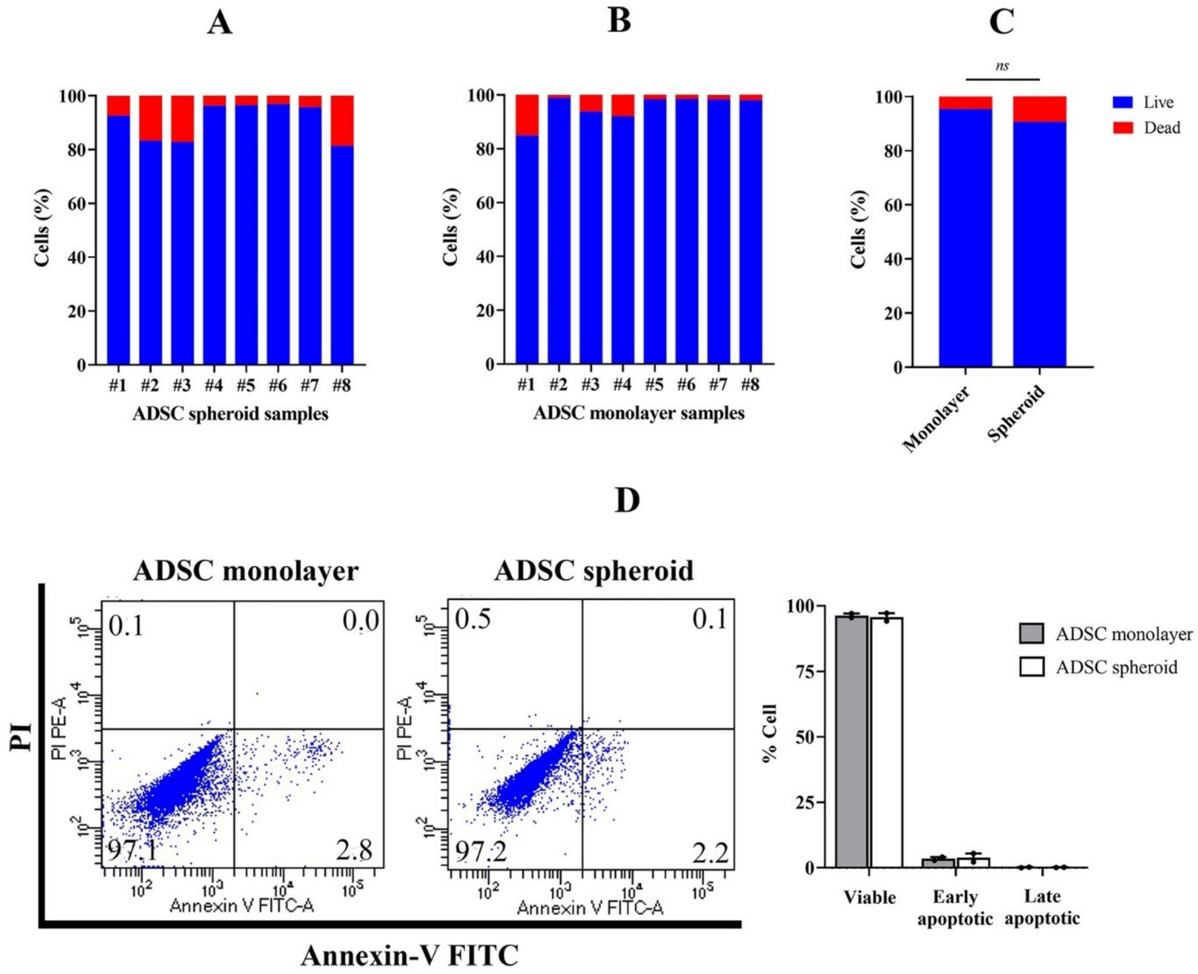

Figure S3. Cell viability analysis. Individual ADSC spheroid (A) and ADSC monolayer (B) were examined for viable cells before cellular extract preparation using trypan blue exclusion assay. The %viability of ADSC derived from spheroid and monolayer was compared (C). The results were presented as mean±SEM (*n*=8). (D) Flow cytometry analysis of Annexin V/PI staining in ADSC monolayer and ADSC spheroid. Graph presents the percentage of viable (Annexin V-, PI-), early apoptotic cell (Annexin V+, PI-), and late apoptotic cell (Annexin V+, PI+) cells of dissociated ADSC spheroid and ADSC monolayer (*n*=2).

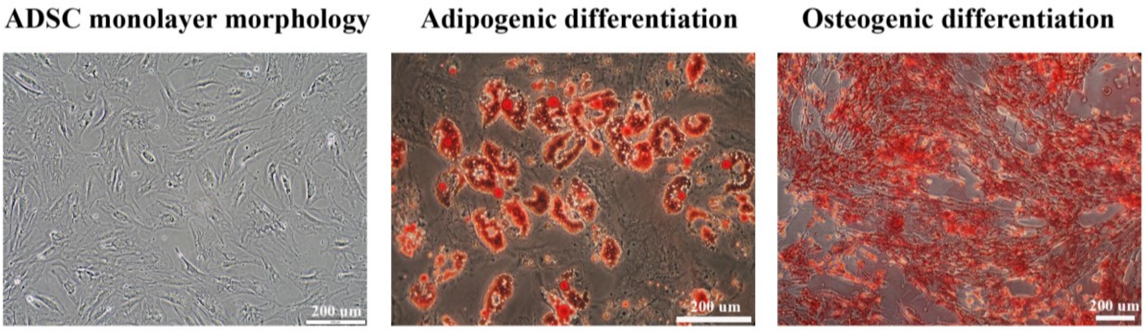

Figure S4 Multilineage differentiation potential of ADSC monolayer. Passage 5th ADSCs cultured as conventional monolayers exhibited fibroblast-like morphology. The cells were able to differentiate toward adipocyte and osteoblast when cultured in adipogenic and osteogenic induction medium for 14-21 days.

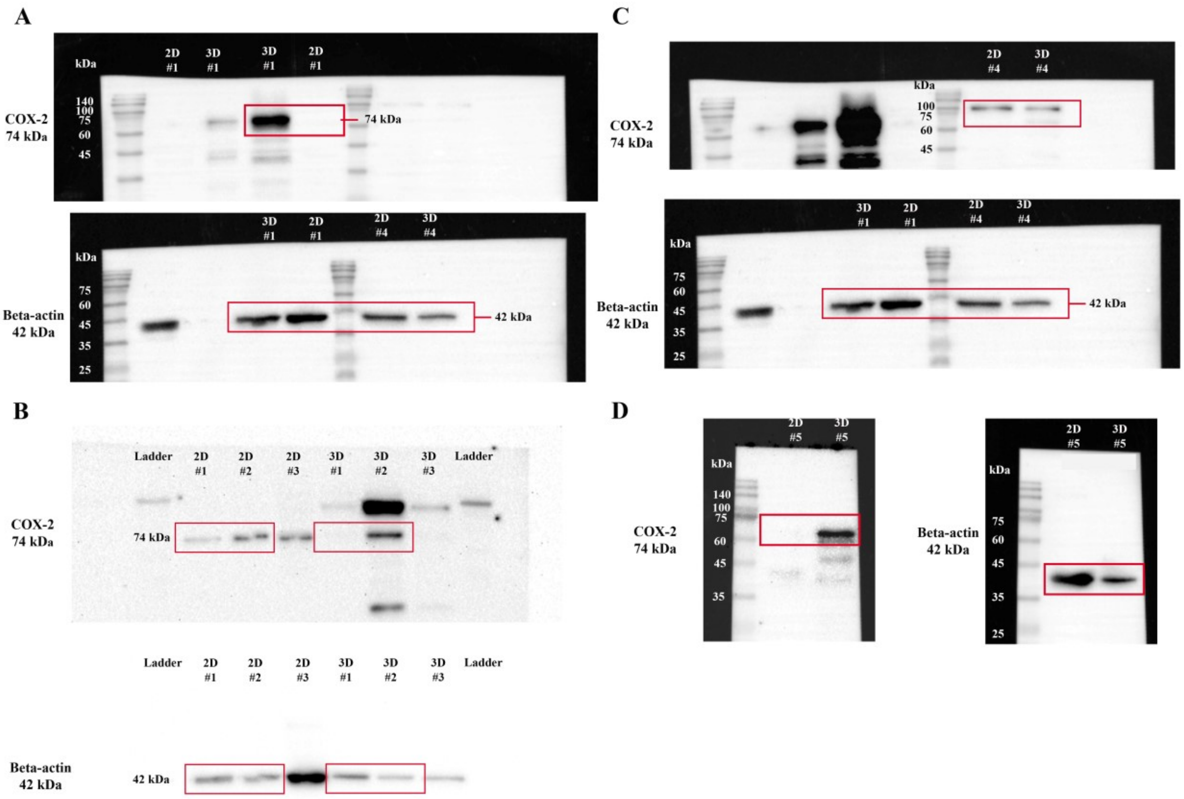

Figure S5: Western blotting analysis. The uncropped blot presents the expression of COX-2 and β-actin from individual 4 ADSC-spheroid extracts compared to ADSC-monolayer extracts which obtained from the same donors (A-D). The red box in (A) represents the sample (#1) that was shown in the article. The band intensity in samples #1, 2, 4, and 5 were measured by using Image Lab™ software (*n*=4).

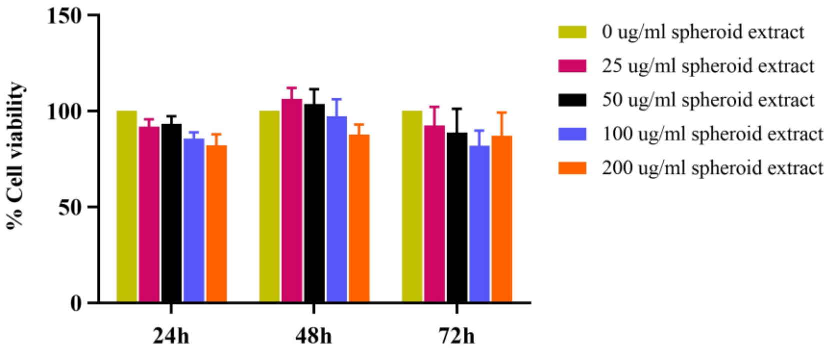

Figure S6 The cytotoxic effects of ADSC spheroid extract on macrophages. The addition of ADSC spheroid extracts (0-200 μg/ml) did not affect PMA-induced macrophage (M0) viability when cultured for 24h, 48h, and 72h, respectively. The results are presented as mean±SEM (*n*=3).
